# Boltz-1 Democratizing Biomolecular Interaction Modeling

**DOI:** 10.1101/2024.11.19.624167

**Published:** 2024-11-20

**Authors:** Jeremy Wohlwend, Gabriele Corso, Saro Passaro, Mateo Reveiz, Ken Leidal, Wojtek Swiderski, Tally Portnoi, Itamar Chinn, Jacob Silterra, Tommi Jaakkola, Regina Barzilay

**Affiliations:** 1 MIT CSAIL; 2 MIT Jameel Clinic; 3 Genesis Research, a part of Genesis Therapeuticss

## Abstract

Understanding biomolecular interactions is fundamental to advancing fields like drug discovery and protein design. In this paper, we introduce Boltz-1, an open-source deep learning model incorporating innovations in model architecture, speed optimization, and data processing achieving AlphaFold3-level accuracy in predicting the 3D structures of biomolecular complexes. Boltz-1 demonstrates a performance on-par with state-of-the-art commercial models on a range of diverse benchmarks, setting a new benchmark for commercially accessible tools in structural biology. By releasing the training and inference code, model weights, datasets, and benchmarks under the MIT open license, we aim to foster global collaboration, accelerate discoveries, and provide a robust platform for advancing biomolecular modeling.

## 1 Overview

Biomolecular interactions drive almost all biological mechanisms, and our ability to understand these interactions guides the development of new therapeutics and the discovery of disease drivers. In 2020, AlphaFold2 [Jumper et al., 2021] demonstrated that deep learning models can reach experimental accuracy for single-chain protein structure prediction on a large class of protein sequences. However, a critical question about modeling biomolecular complexes in 3D space remained open.

In the past few years, the research community has made significant progress toward solving this pivotal problem. In particular, the use of deep generative models has proven to be effective in modeling the interaction between different biomolecules with DiffDock [Corso et al., 2022] showing significant improvements over traditional molecular docking approaches and, most recently, AlphaFold3 [Abramson et al., 2024] reaching unprecedented accuracy in the prediction of arbitrary biomolecular complexes.

In this manuscript, we present Boltz-1, the first fully commercially accessible open-source model reaching AlphaFold3 reported levels of accuracy. By making the training and inference code, model weights, datasets, and benchmarks freely available under the MIT license, we aim to empower researchers, developers, and organizations around the world to experiment, validate, and innovate with Boltz-1. At a high level, Boltz-1 follows the general framework and architecture presented by Abramson et al. [2024], but it also presents several innovations which include:

1. New algorithms to more efficiently and robustly pair MSAs, crop structure at training time, and condition predictions on user-defined binding pockets;
2. Changes to the flow of the representations in the architecture and the diffusion training and inference procedures;
3. Revision of the confidence model both in terms of architectural components as well as the framing of the task as a fine-tuning of the model’s trunk layers.

In the following sections, we detail these changes as well as benchmark the performance of Boltz-1 with other publicly available models. Our experimental results show that Boltz-1 delivers performance on par with the state-of-the-art commercial models on a wide range of structures and metrics.

Given the dynamic nature of this open-source project, this manuscript and its linked GitHub repository^1^ will be regularly updated with improvements from our core team and the community. We aspire for this project and its associated codebase to serve as a catalyst for advancing our understanding of biomolecular interactions and a driver for the design of novel biomolecules.

## 2 Data pipeline

Boltz-1 operates on proteins represented by their amino acid sequence, ligands represented by their smiles strings (and covalent bonds), and nucleic acids represented by their genomic sequence. This input is then augmented by adding multiple sequence alignment (MSA) and predicted molecular conformations. Unlike AlphaFold3, we do not include input templates, due to their limited impact on the performance of large models.

In this section, we first outline how the structural training data, as well as the MSA and conformer, were obtained and describe the curation of our validation and test sets. Then, we describe three important algorithmic developments applied to data curation and augmentation that we find to be critical:

1. A new algorithm to pair MSAs for multimeric protein complexes from taxonomy information (2.3)
2. A unified cropping algorithm that combines the spatial and contiguous cropping strategies used in previous work (2.4)
3. A robust pocket-conditioning algorithm tailored to common use cases (2.5)

### 2.1 Data source and processing

#### PDB structural data

For training we use all PDB structures [Berman et al., 2000] released before 2021–09-30 (same training cut-off date as AlphaFold3) and with a resolution of at least 9Å. We parse the Biological Assembly 1 from these structures from their mmCIF file. For each polymer chain, we use the reference sequence and align it to the residues available in the structure. For ligands, we use the CCD dictionary to create the conformers and to match atoms from the structure. We remove leaving atoms when (1) the ligand is covalently bound and (2) that atom does not appear in the PDB structure. Finally, we follow the same process as AlphaFold3 for data cleaning, which includes the ligand exclusion list, the minimum number of resolved residues, and the removal of clashing chains.

#### MSA and molecular conformers

We construct MSAs for the full PDB data using the colabfold_search tool [Mirdita et al., 2022] (which leverages MMseqs2 [Steinegger and Söding, 2017]), using default parameters (versions: uniref30_2302, colabfold_envdb_202108). We then assign taxonomy labels to all UniRef sequences using the taxonomy annotation provided by UniProt [Consortium, 2015]. For the initial molecular conformers that are provided to the model, we pre-compute a single conformer for all CCD codes using the RDKit’s ETKDGv3 [Wang et al., 2022].

#### Structure prediction training pipeline

We train the structure prediction model (see Section 3.2 for details of the confidence model training) for a total of 68k steps with a batch size of 128. During the first 53k iterations, we use a crop size of 384 tokens and 3456 atoms and draw structures equally from the PDB dataset and the OpenFold distillation dataset (approximately 270K structures, using the MSAs they provided) [Ahdritz et al., 2024]. For the last 15k iterations, we only sampled from the PDB structures and had a crop size of 512 tokens and 4608 atoms. As a comparison AlphaFol3 trained a similar architecture for nearly 150k steps with a batch size of 256, which required approximately four times the computing time. We attribute some of this drastic reduction to the various innovations we detail in the remainder of this section and the next.

### 2.2 Validation and test sets curation

To address the absence of a standardized benchmark for all-atom structures, we are releasing a new PDB split designed to help the community converge on reliable and consistent benchmarks for all-atom structure prediction tasks.

Our training, validation and test splitting strategy largely follows Abramson et al. [2024]. We first cluster the protein sequences in PDB by sequence identity with the command mmseqs easy-cluster … --min-seq-id 0.4 [Hauser et al., 2016]. Then, we select all structures in PDB satisfying the following filters:

1. Initial release date is before 2021–09-30 (exclusive) and 2023–01-13 (inclusive).
2. Resolution is below 4.5Å.
3. All the protein sequences of the chains are not present in any training set clusters (i.e. before 2021–09-30).
4. Either no small-molecule is present, or at least one of the small-molecules exhibits a Tanimoto similarity of 0.8 or less to any small-molecule in the training set. Here, a small-molecule is defined as any non-polymer entity containing more than one heavy atom and not included in the ligand exclusion list.

This yields 1728 structures, which we further refine through the following steps:

1. Retaining all the structures containing RNA or DNA entities. (126 structures)
2. Iteratively adding structures containing small-molecules or ions under the condition that all their protein chains belong to new unseen clusters (330 additional structures)
3. Iteratively adding multimeric structures under the condition that all the protein chains belong to new unseen clusters. These are further filtered by randomly keeping only 50% of the passing structures. (231 additional structures)
4. Iteratively adding monomers under the condition that their chain belongs to a new unseen cluster. These are further randomly filtered out by keeping only 30% of the passing structures. (57 additional structures)

This results in a total of 744 structures. Finally, we retain the structures with at most 1024 residues in the valid protein/RNA/DNA chains, finishing with a total of 553 validation set structures.

The test set is created using the same procedure described above with the following differences: for protein and ligand similarity exclusion we consider all structures released before 2023–01-13 (which include all training and validation sets), we filter to structures released after 2023–01-13 and the final size filter to structures between 100 and 2000 total residues. The resulting final test set size is 593.

### 2.3 Dense MSA pairing algorithm

Multiple sequence alignments uncover amino acids that co-evolved throughout evolution, and therefore are likely close to each other in physical space. However, extracting such signals for protein-protein interactions poses a greater challenge, as most proteins are sequenced or reported individually. To approximate these pairings, researchers have leveraged the taxonomy information frequently associated with sequences. In Algorithm 1, we present a method for pairing MSAs using taxonomy in a manner that preserves MSA density (a critical factor, as model complexity scales linearly with the number of MSA rows) while balancing the trade-off between the signal derived from paired sequences and the sequence redundancy within each chain.

### 2.4 Unified cropping algorithm

In order to efficiently train on complexes with variable size, methods like AlphaFold2 and AlphaFold3 crop the structures during training to a fixed maximum number of atoms, residues, or tokens. The most common techniques to perform such crops are (1) contiguous, where tokens are chosen to be consecutive residues in a biomolecular sequence (or entire molecules), and (2) spatial crops, where tokens are chosen purely depending on their distance from a center token. Each of these two has its advantages and provides different training signals to the models, therefore they are often used in combination as done, for example, by Abramson et al. [2024].

We argue, however, that these are two extremes and it is useful to train the model on a more diverse range of cropping strategies. To this end, we define a new cropping algorithm which directly interpolates between spatial and contiguous strategies. The algorithm, formalized in Algorithm 2, revolves around the definition of neighborhoods, that characterize contiguous portions of sequences of a particular length (or entire non-polymer entities) around a specific token. Neighborhoods are incrementally added to the crop depending on the distance of their central token from the chosen center of the crop. If the size of the neighborhoods is chosen to be zero, this strategy translates into spatial cropping, whereas if the size is half of the maximum token budget, this strategy translates into continuous cropping. In our experiments, we find it beneficial to randomly sample the neighborhood size uniformly between zero and 40 tokens for every training sample.

### 2.5 Robust pocket-conditioning

In many real-world scenarios, researchers have prior knowledge of the protein’s binding pocket. Therefore, it is valuable to enable the model to condition on the pocket information. AlphaFold3 explored pocket-conditioned generation by fine-tuning the model to include an additional token feature for all the pocket-ligand pairs, where the pocket is defined as any residue with heavy atoms within 6Å of the ligand. While effective, this design has some limitations. It requires maintaining two models, one with and one without pocket conditioning, and it assumes the specification of all residues within 6Å. This assumption may not align with realistic scenarios, where users might only know key residues, and the full set of interacting residues is highly dependent on the ligand pose, which is often unknown.

To address these challenges, we implement a different strategy for pocket conditioning, designed to (1) retain a single unified model, (2) ensure robustness to a partial specification of interacting residues, and (3) enable interaction site specification for polymer binders such as proteins or nucleic acids. During training, we incorporate pocket information for a randomly selected binder in 30% of iterations. For these cases, we draw the (maximum) number of pocket residues to reveal from a geometric distribution and randomly select residues from those with at least one heavy atom within 6Å of the binder. This information is then encoded as an additional one-hot token feature provided to the model. The training process for this pocket-conditioning approach is described in detail in Algorithm 3.

## 3 Modeling

For the model architecture and training, we started by reproducing AlphaFold3 as described in the supplementary material of Abramson et al. [2024]. AlphaFold3 is a diffusion model that uses a multi-resolution transformer-based model for the denoising of atom coordinates. The model operates at two levels of resolution: heavy atoms and tokens. Tokens are defined as amino acids for protein chains, nucleic acid bases for RNA and DNA, and individual heavy atoms for other molecules and modified residues or bases.

On top of the denoising transformer, critically, AlphaFold3 also employs a central trunk architecture that is used to initialize tokens’ representations and determine the denoising transformer’s attention pair bias. This trunk is computationally expensive due to its use of token pairs as fundamental “computational token” and its axial attention operations on these pair representations which results in a complexity that scales cubically with the number of input tokens. To make such encoding computationally tractable, the trunk is set to be independent of the specific diffusion time or input structure such that it can be run only once per complex.

Starting from this architecture, we designed and tested a number of potential alternative approaches. In the following sections, we describe the ones that yielded improvements and were therefore adopted into Boltz-1.^2^ Because of the significant computational budget required to train a full-sized model, we tested these changes on a smaller-sized architecture at different points of our development process. We expect our observations to hold for the final full-size model, but cannot present direct ablation studies.

### 3.1 Architectural modifications

#### MSA module

We find it beneficial to reorder the operations performed in the MSAModule (AlphaFold3 Algorithm 8) to better allow the updates on the single and pair representations to feed to one another. In particular, we change the order^3^ of its operations from:

OuterProductMean, PairWeightedAveraging, MSATransition, TriangleUpdates, PairTransition to:

PairWeightedAveraging, MSATransition, OuterProductMean, TriangleUpdates, PairTransition.

Note that OuterProductMean propagates information from the single to the pair representation, so we now allow the single representations learned in the MSATransition to directly propagate to the pair representation.

#### Transformer layer

Abramson et al. [2024] presents an unusual order of operations in their DiffusionTransformer layers where hidden representations are updated as (AlphaFold3 Algorithm 23):

a ← AttentionPairBias(a) + ConditionedTransitionBlock(a).

This has two issues (1) it lacks residual connections that may make backpropagation more complex and (2) it does not allow for the transformation learned in the AttentionPairBias to be fed in the ConditionedTransitionBlock at the same block. We found it to be beneficial to apply the following transformation order:

a ← a + AttentionPairBias(a)

a ← a + ConditionedTransitionBlock(a).

### 3.2 Training and inference procedures

#### Kabsch diffusion interpolation

A key change between AlphaFold2 and AlphaFold3 was the non-equivariance of the denoising model of AlphaFold3 (compared to the equivariant IPA-based structure module of AlphaFold2) to rotations and translations. To encourage the robustness of the denoising model to such transformations their input is randomly translated and rotated before the denoising at training and inference times. To further reduce the variance of the denoising loss with respect to these variations, Abramson et al. [2024] use a rigid alignment between the predicted denoising coordinates and the true coordinates before computing the MSE loss.

However, we argue that on its own this procedure is theoretically problematic. One can define simple functions that would achieve zero rigid aligned MSE loss during training, but completely fail to sample realistic poses at inference time. For example, consider a model trying to fit a given structure with coordinates x. Let’s assume that for any noised structure within some reasonable noising perturbation (e.g. $\text{Δ}=10\sigma_{t}$), the model always predicts x:

$${\hat{x}}_{t}^{\text{denoised}}=f\left({\hat{x}}_{t}^{\text{noisy}},t\right)=f\left(x*R^{⊤}+T+\epsilon_{t},t\right)=\{\begin{array}{cc} x & \text{if}\;\left\|\epsilon_{t}\right\|<10\sigma_{t},\\ 0 & \text{otherwise}. \end{array}$$

where R and T are respectively a random rotation matrix and a random translation vector, $\epsilon_{t}$ and $\sigma_{t}$ represent respectively the random noise and noise standard deviation for some diffusion time t. This model will have a loss approaching zero during training (one will never sample something beyond 10 standard deviations, and one could make this Δ arbitrarily large). However, when used at inference time, this model will consistently go out of distribution (and therefore predict a zero vector). This is because at low noise levels the interpolation between the current randomly rotated ${\hat{x}}_{t}^{\text{noisy}}$ and the predicted ${\hat{x}}_{t}^{\text{denoised}}$ may lead to a pose ${\hat{x}}_{t-\text{Δ}t}$ that is very far from x and will fall beyond the 10 $\sigma_{t}$ mark. Figure 2 shows a graphical representation of this issue.

We overcome this issue by adding a rigid alignment with Kabsch algorithm after every step during the inference procedure before ${\hat{x}}_{t}^{\text{noisy}}$ and ${\hat{x}}_{t}^{\text{denoised}}$ are interpolated (see Figure 2 for a visual explanation). Informally, our diffusion interpolation operates on the minimal projection between noisy and denoised structures, guaranteeing, under the assumption of a Dirac distribution, that the interpolated structure is more similar to the denoised sample than the noisy structure. Empirically, we note that this change to the reverse diffusion has a bigger effect when training models on subsets of the full data where the model is more likely to overfit, on the other hand, the final Boltz-1 seems to largely denoising close to the projection making the Kapsch alignment not critical.

#### Diffusion loss weighting

For the weighting of the diffusion loss we use $\left({\hat{t}}^{2}+\sigma_{\text{data}}^{2}\right)/{\left(\hat{t}\times \sigma_{\text{data}}\right)}^{2}$ in line with the EDM framework [Karras et al., 2022], rather than $\left({\hat{t}}^{2}+\sigma_{\text{data}}^{2}\right)/{\left(\hat{t}+\sigma_{\text{data}}\right)}^{2}$ (AlphaFold3 Section 3.7.1 Eq. 6).

### 3.3 Confidence model

AlphaFold3 trains the confidence model alongside the trunk and denoising models while, however, cutting all the gradients going from the confidence task to the rest of the model. Instead, training structure prediction and confidence models separately allowed us to disentangle experiments on each component and make several important improvements to the confidence prediction task.

In AlphaFold3 the architecture of the confidence model is composed of four PairFormer layers that take as input the final single and pair token representations from the model trunk as well as an encoding of the token pairwise distances predicted by the reverse diffusion. These four layers are followed by linear projections trained to predict whether each atom is resolved in the crystal structure, per-atom LDDT and per-token pair PAE and PDE.

#### Trunk architecture and initialization

We noticed that, at a high level, the input-output composition of the confidence model is similar to that of the trunk. The trunk also takes as input its own final representations (through recycling) and outputs expressive representations used by the denoising model. Therefore, inspired by the way that researchers in the large language model community have been training reward models by fine-tuning the “trunk” of their pretrained generative models [Touvron et al., 2023], we define the architecture of our confidence model to contain all the components of the trunk and initialize its representation to the trained trunk weights. Hence, our confidence model presents an AtomAttentionEncoder, an MSAModule, and a PairFormerModule with 48 layers. In addition, we still integrate the predicted conformation as an encoding of the pairwise token distance matrix and decode the confidence with linear layers on the final PairFormer representation.

#### Diffusion model features

We feed to the confidence model not only the representations coming from the trunk but also a learned aggregation of the final token representation at each reverse diffusion step. These representations are aggregated through the reverse diffusion trajectory with a time-conditioned recurrent block and then fed concatenated to the trunk token-level features at the start of the confidence model. We further modify the way that token-level features are fed to the pairwise representations adding an element-wise multiplication of linearly transformed token-level features.

#### Overall procedure and training

We detail our new full inference procedure in Algorithm 4 and provide a schematic representation in Figure 3. To train the confidence model we initialize all the components borrowed by the trunk to the final trunk weights (from the exponentially moving average) and initialize the weights of all the other components of the network randomly but with zeroed final layers not to perturb initial rich representation from the pretrained weights.

Note: at the time of the release of the current manuscript, the large version of the confidence model is finishing training so it is not yet available in our GitHub repository. We will update the repository with the confidence model as well as the results section in this manuscript in the coming days once training has converged.

### 3.4 Optimizations

Below we summarize some computational techniques we use to speed up and/or reduce the memory consumption of the model. For details on the implementation of each of these, please refer to our code repository.

#### Sequence-local atom representation

The AtomAttentionEncoder and AtomAttentionDecoder include a pair-biased transformer on the representations of every atom. In particular, the attention of these transformers is sequence-local: blocks of 32 atoms only attend to the 128 atoms that are closest to them in sequence space. We developed a GPU-efficient implementation of the sequence-local attention precomputing a mapping (performed in blocks of 16 tokens) to the key and query sequence embeddings for each 32 key tokens block. The attention is then performed in parallel in each 32×128 block achieving a block sparse attention with dense matrices.

#### Attention bias sharing and caching

At a high level the denoising model must be run repeatedly for every diffusion timestep for every separate sample we take, while the trunk can be run once and its representation fed to all those denoising model passes.

The most expensive components of the denoising model are represented by the computation of the attention pair bias for the token and atom transformers. However, by examining their computational graph, we find that these elements do not depend either on the particular input structure given to the denoising model or the diffusion timestep. In particular, these elements are: the attention bias of all the transformer layers in the AtomAttentionEncoder, AtomAttentionDecoder, and DiffusionTransformer, and the intermediate single and pairwise atom representations of the AtomAttentionEncoder. Therefore, we can also run these components once and share them across all the samples and the entirety of the reverse diffusion trajectory, significantly reducing the computational cost of the reverse diffusion at the cost of storing these representations and biases in memory.

#### Greedy symmetry correction

During validation and confidence model training, the optimal alignment between the ground truth and predicted structure must be determined, accounting for permutations in the order of identical chains or symmetric atoms within those chains. Because the number of possible perturbations grows exponentially with the size of the complex, considering all of them is computationally unfeasible.

We devise the following procedure to perform an approximate, yet effective, atom matching. This operates in a hierarchical way searching (1) the optimal assignment of chains, and, then, (2) assuming chain assignment, to select atom perturbations greedily for every ligand or residue. For the first, for each symmetric chain, we compute the resulting global LDDT without changing any inner atom assignment. For the second step, iteratively for every ligand, amino acid, or nucleotide basis (one at a time), we find the perturbation of that ligand the most improves the global LDDT and greedily apply it.

Note that because the LDDT between pairs of elements that were not affected by the perturbation does not change, one can implement the test in the last step very efficiently only looking at the specific rows and columns of the distance matrix that change. In practice, we limit the number of perturbations of the chain assignment we consider to 100 and the perturbations of the atoms of each ligand to 1000.

## 4 Results

We evaluate the performance of the model on two benchmarks: the diverse test set of recent PDB structures that we cured as discussed in Section 2.2, and CASP15, the last community-wide protein structure prediction competition where for the first time RNA and ligand structures were also evaluated [Das et al., 2023, Robin et al., 2023]. Both these benchmarks contain a very diverse set of structures including protein complexes, nucleic acids, and small-molecule, making them great testbeds for the assessment of models, such as Boltz-1, capable of predicting the structure of arbitrary biomolecules.

### Benchmarks

For CASP15, we extract all the competition targets with the following filters: (1) they were not canceled from the competition, (2) they have an associated PDB id to obtain the ground truth crystal structure, (3) the number of chains in the stochiometry information matches the number of provided chains, (4) the total number of residues with below 2000. This leaves a total of 76 structures. For our test set, we remove structures with covalently bounded ligands because the current version of the Chai-1 public repository does not provide a way to set these. Finally, for both datasets, we remove structures that go out of memory for either of the two methods on A100 80GB GPUs. After these steps, we are left to evaluate 72 structures for CASP15 and 520 structures for the test set.

### Baselines

We evaluate our performance against Chai-1 [Chai et al., 2024], the first replication of AlphaFold3 that was recently released under an exclusive commercial license and has been shown to match AlphaFold3 results across several benchmarks. At the time of writing this manuscript, AlphaFold3 inference code has just been released, we have requested but not yet received the model weights. We ran the Chai-1 model using the chai_lab package version 0.2.1. The model was run with 200 sampling steps and 10 recycling rounds, and producing 5 outputs, matching our model. We also used the same pre-computed MSA’s up to 16384 sequences. Since Chai-1 requires annotating the source of the sequences, we annotated all Uniref sequences with the uniref90 label and all other sequences with the bfd_uniclust label. We briefly experimented with alternative labelings but did not find these to impact the model substantially.

### Evaluation criteria

We consider several well-established metrics to evaluate the performance of the models on these very diverse sets of biomolecules and structures. In particular, we compute:

1. The median all-atom LDDT: measuring accuracy of local structures across all biomolecules;
2. The median TM-score: measuring global structure quality;
3. The average DockQ success rates, i.e. the proportion of predictions with DockQ > 0.23, which measures the number of good protein-protein interactions predicted;
4. The median protein-ligand interface LDDT: measuring the quality of the ligand and pocket predicted interactions, official CASP15 metric to evaluate the ligand category;
5. The proportion of ligands with a pocket-aligned RMSD below 2Å: a widely adopted measure of molecular docking accuracy.

All metrics were computed using OpenStructure [Biasini et al., 2013]. LDDT-PLI, DockQ and ligand RMSD success rates are computed over all the different protein-protein and protein-ligand interfaces. Following a similar format to that used in CASP and to allow a fair comparison of the methods, we run both Chai-1 and Boltz-1 to generate 5 samples and evaluate the top prediction out of the 5 for every metric.

## Results

We report the performance of Chai-1 and Boltz-1 in Figure 4. The models show comparable results in terms of median LDDT and median TM scores across both CASP15 and the test set, indicating similar accuracy in predicting general biomolecular structures. A similar trend is observed specifically for CASP15 RNA targets, where Chai-1 achieves a median LDDT of 0.41 and a median TM score of 0.31, compared to Boltz-1’s 0.54 and 0.31, respectively.

**Figure 1:**
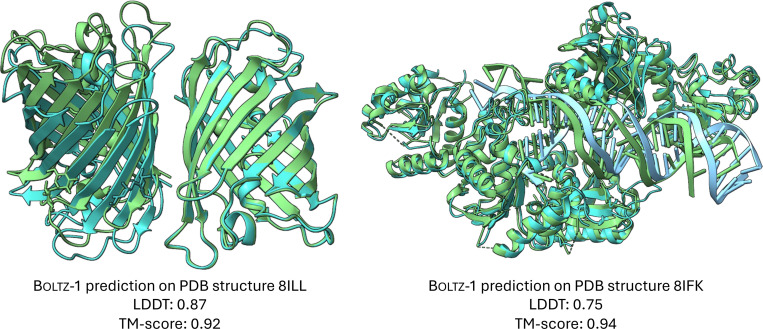
Example predictions of Boltz-1 on targets from the test set.

**Figure 2:**
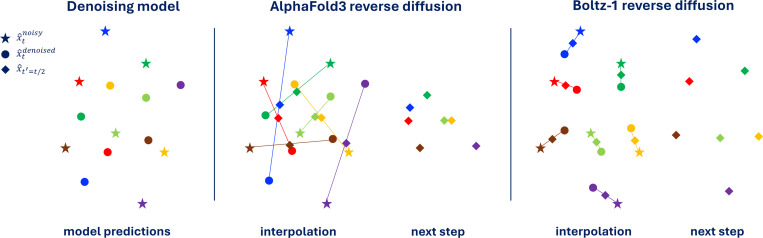
2D representation of the difference between AlphaFold3 reverse diffusion and Boltz-1 reverse diffusion with our Kapsch interpolation. Colors indicate correspondence between different points. Even though the prediction of the denoising model is “perfect” according to the aligned MSE loss, the unaligned interpolation may lead to poor structures fed to the next reverse diffusion step.

**Figure 3:**
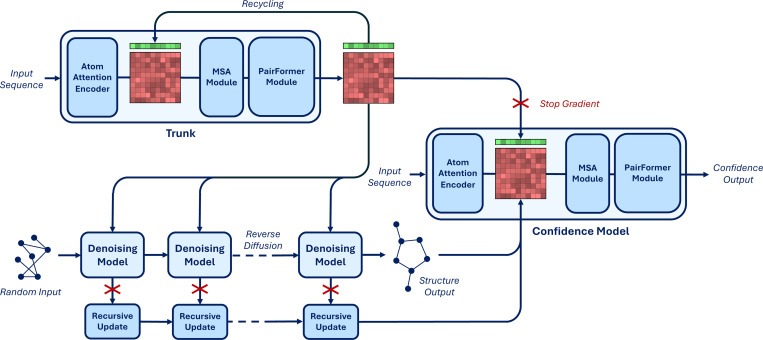
Diagram of the architecture of Boltz-1. The critical difference with AlphaFold3 lies in the confidence model, which now not only has a PairFormerModule but follows a full trunk composition and is fed features coming from the denoising model through the recursive updates.

**Figure 4:**
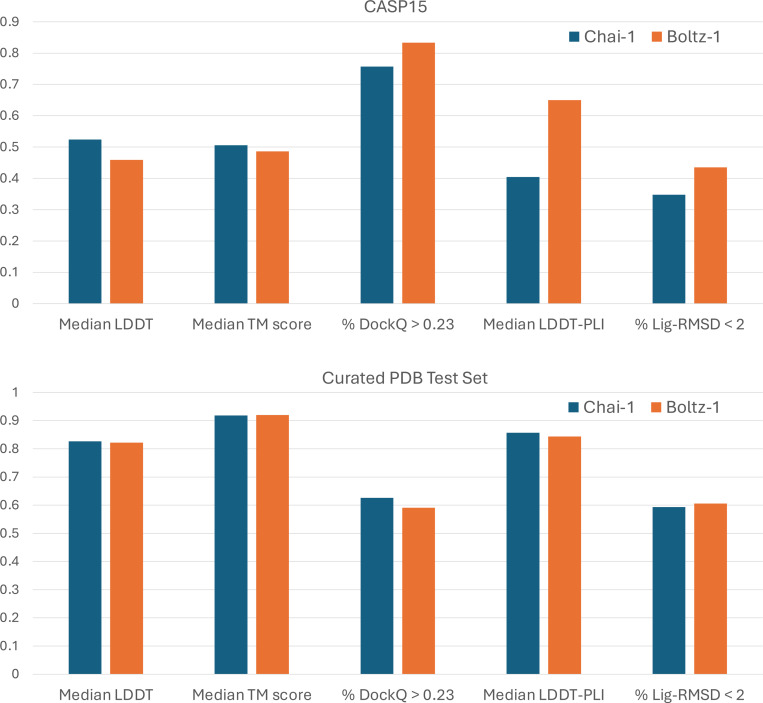
Visual summary of the performance of Chai-1 and Boltz-1 on the CASP15 benchmark and the test set.

For protein-protein interactions, the performance of both methods is also aligned. Chai-1 slightly outperforms Boltz-1 on the test set, while Boltz-1 performs better on CASP15. In the protein-ligand metrics, the two models achieve comparable results on the test set, but Boltz-1 demonstrates a notable advantage on CASP15. This is particularly encouraging given that CASP15 is widely regarded as a challenging dataset for protein-ligand prediction methods [Morehead et al., 2024], owing to its demand for generalization and the modeling of multiple interacting ligands across many targets. However, it is important to note that CASP15 includes only 15 ligand-related targets, encompassing 58 ligands in total, which may limit the robustness of these findings in demonstrating a definitive performance improvement for Boltz-1. Finally, we present in Figure 1 two examples of hard targets from the test set where Boltz-1 performed remarkably well with TM scores above 90%.

## 5 Limitations

**Figure 5:**
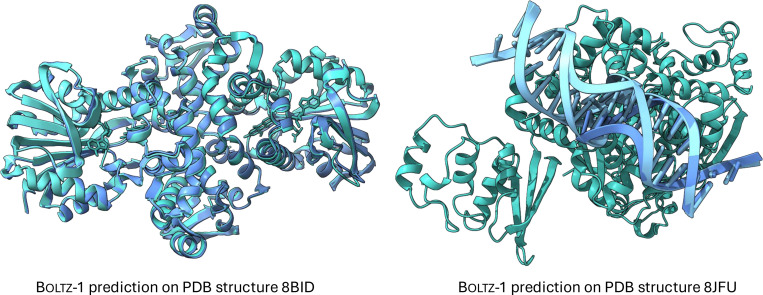
Examples of Boltz-1 predicting chains overlapped on top of one another. On structure 8BID Boltz-1 predicts two pairs of protein chains almost completely overlapped with one another, while on structure 8JFU two pairs of two DNA chains are overlapped.

A visual inspection of several predictions from Boltz-1 revealed instances of hallucinations in the model’s outputs. The most prominent type of hallucination involved the placement of entire chains directly on top of one another. These occurrences exhibited two common patterns: the first involved identical polymer chains in large complexes (examples are shown in Figure 5), while the second involved similar ligands that shared a common substructure.

We propose several hypotheses to explain these patterns:

1. Overlapping chains and ligands in the data: Although our data processing pipeline removed overlapping polymer chains, we did not eliminate overlapping ligands. Upon closer inspection, we found that several examples in the PDB database report overlapping ligands within the same structure, potentially to represent alternative binding molecules or reactions (e.g., PDB ID 7X9K). Such structures in the training set likely introduce misleading learning signals.
2. Insufficient training crop sizes: Due to computational limitations, we trained the model using crop sizes of 384 and 512 tokens, which are significantly smaller than many of the complex structures where these issues were observed. This likely hindered the model’s ability to capture sufficient spatial context during training.

We leave further exploration of alternative training or fine-tuning strategies to mitigate these issues to future iterations of the model. By making the model and its code openly available, we hope to inspire the community to investigate additional limitations and propose innovative solutions to enhance its performance.

## 6 Conclusion

We introduced Boltz-1, the first fully commercially accessible open-source model to achieve AlphaFold3-level accuracy in predicting the 3D structures of biomolecular complexes. To accomplish this, we replicated and expanded upon the AlphaFold3 technical report, incorporating several innovations in architecture, data curation, training, and inference processes. We empirically validated Boltz-1 against Chai-1, the current state-of-the-art structure prediction method, demonstrating comparable performance on both a diverse test set and the CASP15 benchmark.

The open-source release of Boltz-1 represents a significant step forward in democratizing access to advanced biomolecular modeling tools. By freely providing the training and inference code, model weights, and datasets under the MIT license, we aim to enable researchers and organizations to experiment and innovate using Boltz-1. We envision Boltz-1 as a foundational platform for researchers to build upon, fostering collaboration to advance our collective understanding of biomolecular interactions and accelerating breakthroughs in drug design, structural biology, and beyond.

## Figures and Tables

**Algorithm 1:** Dense MSA Pairing

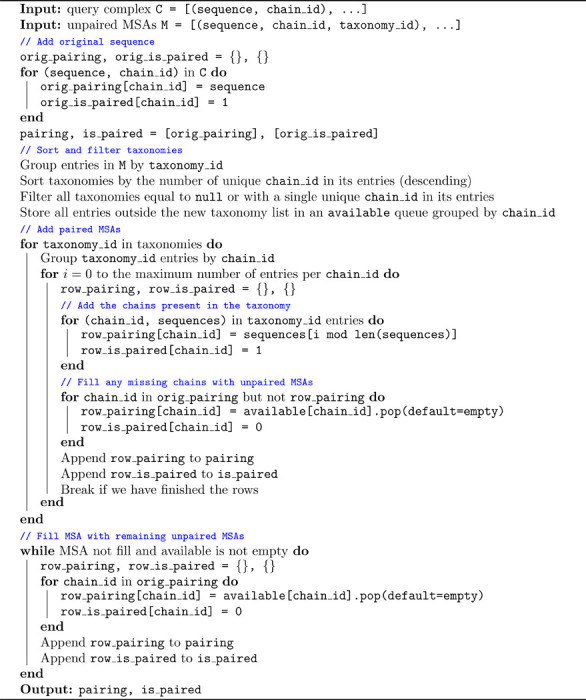

**Algorithm 2:** Unified Cropping

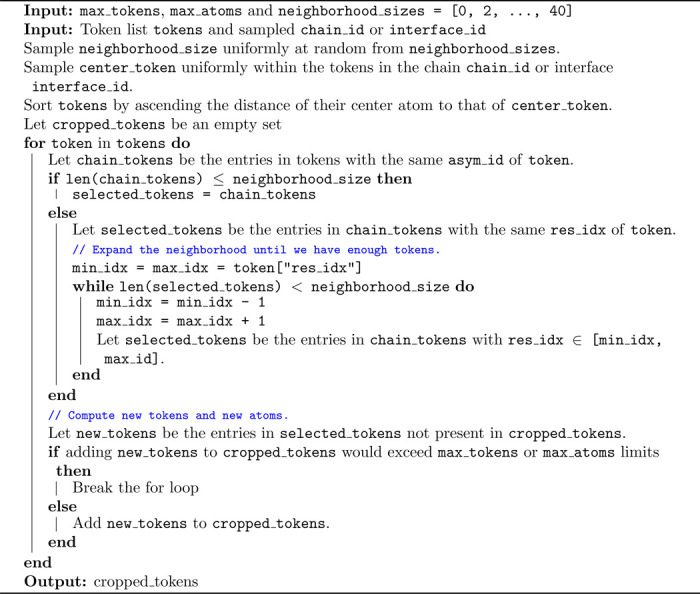

**Algorithm 3:** Robust pocket-conditioning

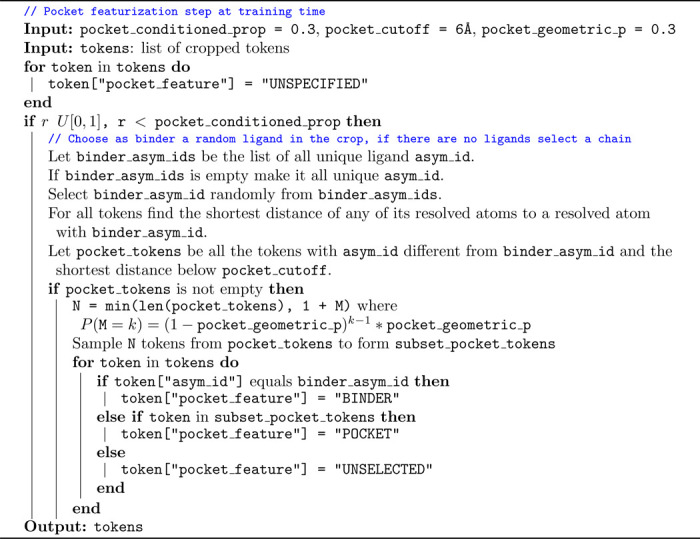

**Algorithm 4:** Confidence model

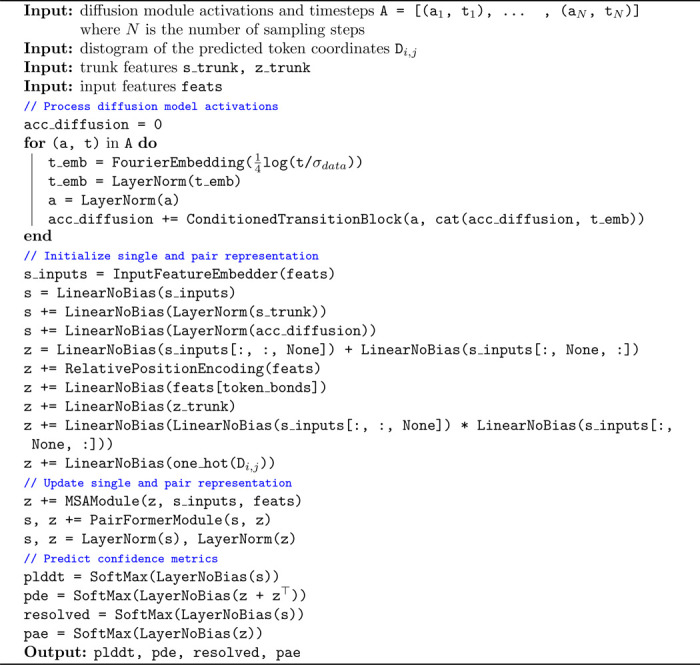

## Footnotes

1
https://github.com/jwohlwend/boltz

2 Some of these differences may simply be the result of reporting mistakes in the current version of the original manuscript from Abramson et al. [2024], as reported .

3 We note that a similar strategy was also concurrently noticed by https://github.com/Ligo-Biosciences/AlphaFold3.
